# Presumed Very Late Relapse of Lepromatous Leprosy 35 Years After Treatment: A Diagnostic Challenge in a Non-endemic Setting

**DOI:** 10.7759/cureus.101651

**Published:** 2026-01-15

**Authors:** Ana Maria Carvalho, Pedro Sá Almeida, Rita G Magalhães, Patrícia Clara, João Silva, Joana Cunha

**Affiliations:** 1 Internal Medicine, Unidade Local de Saúde de Trás-os-Montes e Alto Douro, Chaves, PRT; 2 Internal Medicine, Unidade Local de Saúde de Trás-os-Montes e Alto Douro, Vila Real, PRT; 3 Internal Medicine, Centro Hospitalar Universitário de São João, Porto, PRT

**Keywords:** leprosy, mycobacterium leprae, neglected tropical diseases, peripheral neuropathy, relapse

## Abstract

Leprosy is a chronic infectious disease caused by *Mycobacterium leprae* that primarily affects the skin and peripheral nerves. Although eliminated as a public health problem in many countries, it remains an important neglected tropical disease, with more than 174,000 new cases reported worldwide in 2023, predominantly in endemic regions such as India, Brazil, and Indonesia. We report a rare case of a presumed very late relapse of lepromatous leprosy occurring 35 years after treatment completion in a patient from a non-endemic European setting. The patient presented with progressive sensorimotor neuropathy, progressive deformities, and a chronic non-healing plantar ulcer. Historical records confirmed biopsy-proven lepromatous leprosy at the initial diagnosis, with documented cure following multidrug therapy. Current evaluation revealed the molecular detection of *M. leprae* in a skin biopsy, although histopathological features of active disease were not demonstrated. Given the absence of epidemiological risk factors for reinfection, the long disease-free interval, and the overall clinical and microbiological context, a very late relapse was considered the most plausible explanation. This case highlights the diagnostic challenges of leprosy in non-endemic settings and underscores the importance of long-term clinical vigilance and awareness of late complications.

## Introduction

Leprosy, or Hansen's disease, is a chronic granulomatous infection caused by *Mycobacterium leprae* that primarily affects the skin and peripheral nerves [1]. Although global prevalence has markedly declined since the introduction of multidrug therapy, new cases continue to emerge in endemic areas and, occasionally, in non-endemic countries through migration [2,3]. According to the World Health Organization, more than 174,000 new cases of leprosy were reported worldwide in 2023, with the highest burdens in India, Brazil, and Indonesia [1]. Portugal achieved elimination as a public health problem in the 1990s, and only sporadic cases (<5 per year) have been reported since, mainly among migrants from endemic regions [4,5]. Despite the overall reduction in disease burden, very late relapse remains a recognized but uncommon phenomenon, particularly in patients treated decades ago under historical diagnostic and therapeutic criteria. This context reinforces the need to maintain clinical awareness, even in regions where the disease is rare.

## Case presentation

A 58-year-old man was originally diagnosed with lepromatous leprosy at the age of 23 in northern Portugal, during a locally reported cluster associated with mining activity. As leprosy is a notifiable disease in Portugal, at the time of the initial diagnosis, the patient was evaluated by a specialized multidisciplinary team after presenting with a left plantar ulcer, within the appropriate epidemiological context of the outbreak. The diagnosis was confirmed by biopsy of the left sural nerve, according to official medical records obtained from that period.

The patient completed the standard multidrug therapy recommended at the time under directly observed treatment at the healthcare unit and was subsequently declared cured based on the clinical and bacteriological criteria in use during that period. At the time of cure declaration, the initial left plantar ulcer had fully resolved, and no additional cutaneous or musculoskeletal deformities were documented in the available records.

Thirty-five years later, he was referred to our department for the evaluation of a new left plantar ulcer with chronic evolution, located at a different plantar site from the initial lesion. The patient reported the onset of progressive lower limb pain approximately five years prior to presentation. However, due to stigma associated with his previous diagnosis of leprosy, he avoided seeking medical care and lived in social isolation, relying on subsistence agriculture. During this period, he adapted to the gradual development of neuropathic symptoms, new cutaneous lesions, and functional impairment. He ultimately sought medical attention after the plantar ulcer failed to heal despite home-based care.

**Figure 1:**
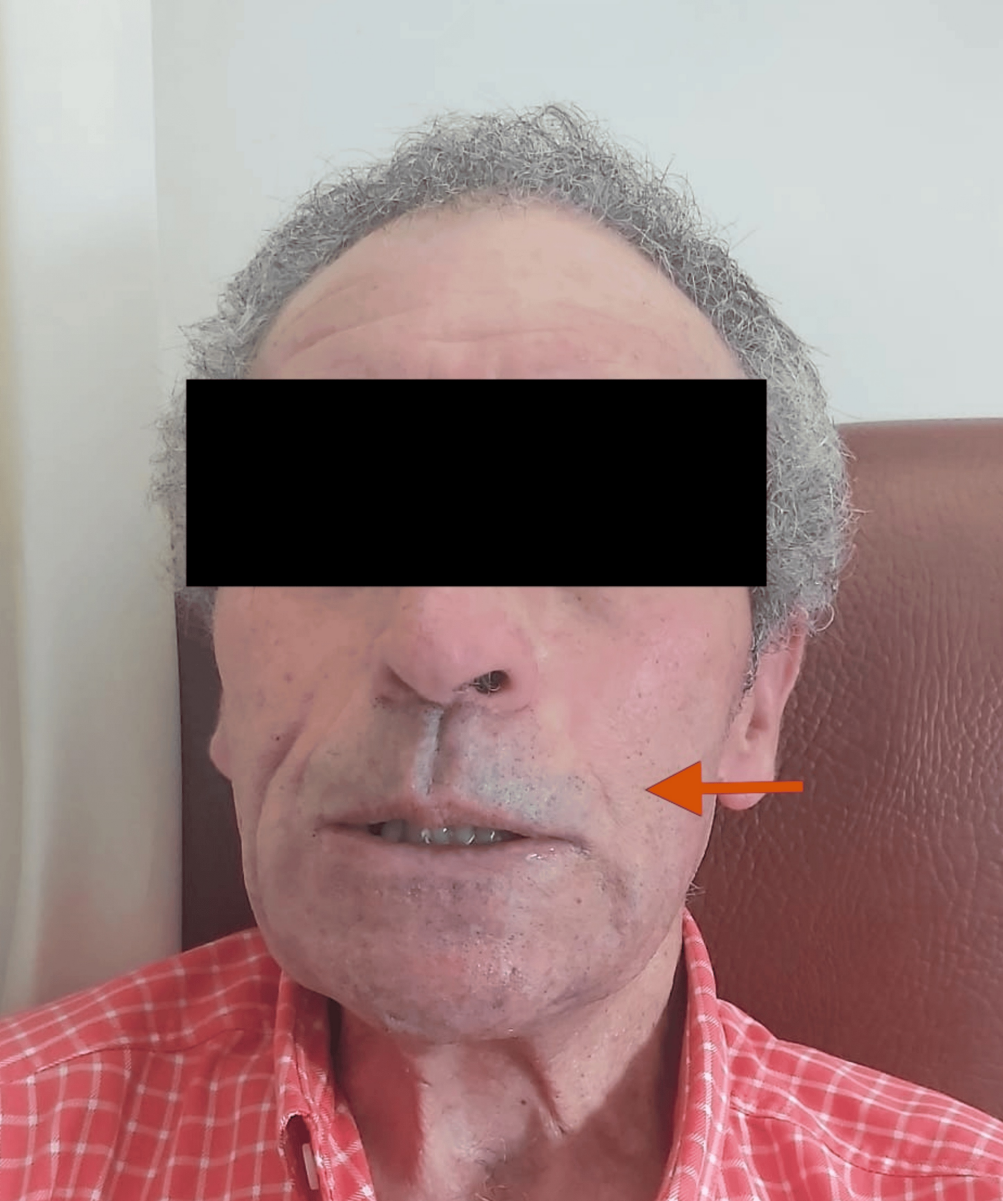
Facial hypomimia and left-sided facial paresis (arrow), consistent with the involvement of the facial nerve in Hansen's disease

**Figure 2:**
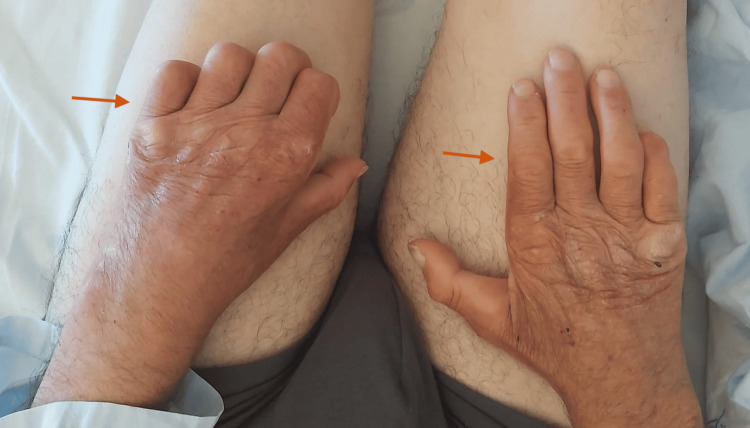
Bilateral hand deformities with clawing of the fingers (arrows), reflecting severe sensorimotor neuropathy

**Figure 3:**
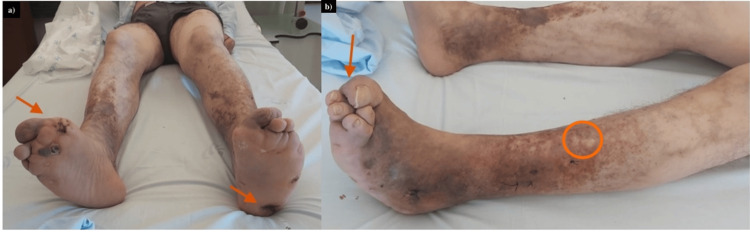
Bilateral plantar ulcers and toe deformities secondary to sensory loss and neuropathy (a) Bilateral plantar ulcers (arrows). (b) Toe deformities (arrows) and hypopigmented skin lesions (circles), consistent with chronic neuropathic and cutaneous involvement.

On examination, we observed facial hypomimia and left-sided facial paresis (Figure 1). There was evidence of progressive bilateral involvement, with hypopigmented and erythematous skin lesions, sensory loss, and deformities of both hands and feet, including clawing of the fingers (Figure 2). The hypopigmented skin lesions and bilateral plantar ulcers are best appreciated in Figure 3, which also demonstrates associated toe deformities.

Alternative causes of peripheral polyneuropathy and secondary ulcer infection were systematically evaluated. The patient had no history of diabetes mellitus, and glycemic control was normal, with hemoglobin A1c within the reference range. There was no history of alcohol abuse, nutritional deficiencies, or exposure to neurotoxic agents. Clinical examination revealed no features suggestive of Parkinson's disease, such as resting tremor, rigidity, or bradykinesia, despite the presence of facial hypomimia. Microbiological evaluation of the plantar ulcer, including bacterial and fungal cultures, was negative, excluding secondary infection.

Skin biopsy with molecular analysis was positive for *M. leprae*. Electromyography revealed severe chronic axonal sensorimotor polyneuropathy affecting both upper and lower limbs, consistent with Hansen's neuropathy. Ocular motility and extraocular muscles were spared. Taken together, the clinical, laboratory, microbiological, and electrophysiological findings supported a neuropathy most consistent with long-standing Hansen's disease. Glucose-6-phosphate dehydrogenase activity was normal, and multidrug therapy with dapsone, rifampicin, and clofazimine was reinitiated, which the patient continues to tolerate well.

Given the absence of active transmission risk, isolation measures were not required.

## Discussion

Leprosy relapse is a rare but recognized phenomenon that may occur years after the completion of multidrug therapy [6,7]. Very late relapse, occurring more than three decades after treatment, is exceptionally uncommon [8]. In lepromatous leprosy, where the bacillary load is high, relapse has been attributed to the persistence of dormant bacilli within the peripheral nerves or skin macrophages, with reactivation occurring long after apparent cure [9].

In the present case, the clinical findings are most consistent with a presumed very late relapse of lepromatous leprosy. The initial diagnosis was histopathologically confirmed by sural nerve biopsy, and the patient completed multidrug therapy under directly observed conditions, with the documented resolution of the initial plantar ulcer at the time of cure. Importantly, there were no medical records documenting residual neurological deficits, deformities, or chronic ulcers following the completion of treatment, suggesting a prolonged period of clinical stability.

After this long disease-free interval, the patient developed new and progressive clinical manifestations, including sensorimotor neuropathy, deformities of the hands and feet, and a chronic non-healing plantar ulcer at a different site. The emergence of these findings decades after documented cure argues against static post-treatment sequelae and supports a new disease process occurring long after initial treatment.

Reinfection was carefully considered in the differential diagnosis but was deemed unlikely given the absence of reported leprosy outbreaks in Portugal in recent decades, the patient's long-term residence in a non-endemic region, lack of travel to endemic countries, absence of known contact with active cases, and prolonged social isolation. Although direct molecular comparison between the initial and current isolates was not possible, the epidemiological context strongly supports relapse as the most plausible explanation rather than reinfection.

Molecular detection of *M. leprae *in the current skin biopsy further supports ongoing disease activity, although polymerase chain reaction (PCR) positivity alone does not distinguish viable from dormant bacilli. PCR-based techniques are highly sensitive and can detect low bacillary loads, particularly in cases with atypical presentation, partial treatment, or early reactivation. While it is acknowledged that molecular positivity may also reflect the persistence of nonviable organisms, the integration of these findings with the patient's compatible clinical evolution, progressive neurological deterioration, emergence of new cutaneous lesions, and development of a new non-healing plantar ulcer supports the interpretation of active disease rather than the mere persistence of dormant bacilli. Histopathological features classically associated with active lepromatous disease were not demonstrated on the most recent biopsy, which represents an important limitation and precludes the definitive confirmation of relapse [10]. Nevertheless, when interpreted in conjunction with the documented history of biopsy-proven lepromatous leprosy, completed treatment, long disease-free interval without documented sequelae, absence of epidemiological risk for reinfection, and compatible clinical evolution, the findings support presumed very late relapse as the most coherent interpretation.

From a public health perspective, this case highlights the need for continued clinical vigilance even decades after the treatment of leprosy. In non-endemic European settings, late relapse should be considered in patients with a remote history of leprosy who present with new-onset neuropathy, deformities, or non-healing plantar ulcers. Awareness of this possibility is essential to avoid diagnostic delay and prevent avoidable morbidity.

## Conclusions

This case illustrates a rare and presumed very late relapse of lepromatous leprosy occurring 35 years after documented cure in a non-endemic European setting. The development of new neuropathic manifestations, progressive deformities, and a non-healing plantar ulcer after decades of clinical stability highlights the diagnostic challenges of leprosy in low-incidence regions. Clinicians should remain alert to the possibility of very late relapse in patients with a remote history of leprosy who present with new-onset neuropathy or difficult-to-heal plantar ulcers in order to avoid diagnostic delay and prevent avoidable morbidity.
